# Fulminant hepatic failure and acute renal failure as manifestations of concurrent Q fever and cytomegalovirus infection: a case report

**DOI:** 10.1186/s12879-014-0651-8

**Published:** 2014-12-09

**Authors:** Jin-Yi Hsu, Chen-Chi Tsai, Kuo-Chih Tseng

**Affiliations:** School of Medicine, Tzuchi University, Hualien, Taiwan; Department of Internal Medicine, Dalin Tzu Chi Hospital, Buddhist Tzu Chi Medical Foundation, No 2, Ming-Shen Road, Dalin Town, 622 Chia-Yi County Taiwan

**Keywords:** Acute renal failure, Cytomegalovirus, Fulminant hepatic failure, Q fever

## Abstract

**Background:**

*Coxiella burnetii* is an obligate bacterial pathogen that causes Q fever. Cytomegalovirus (CMV) commonly exists as a latent infection in healthy people. Co-infection with both pathogens is rare.

**Case presentation:**

We report an immunocompetent 53-year-old male farmer who presented with fulminant hepatic failure and acute renal failure. Empiric antibiotic treatment with intravenous penicillin G and levofloxacin were given, but hepatic and renal functions continued to deteriorate. A subsequent test of serum immunoglobulin M was positive for CMV, and administration of gancyclovir led to gradual recovery. A diagnosis of acute Q fever was confirmed by indirect immunofluorescence assay (IFA) on paired serum samples to demonstrate a significant rise in antibody titers. Antibiotic treatment was adjusted accordingly.

**Conclusion:**

CMV co-infection should be considered in patients with acute Q fever when they do not respond to standard antimicrobial agents.

**Electronic supplementary material:**

The online version of this article (doi:10.1186/s12879-014-0651-8) contains supplementary material, which is available to authorized users.

## Background

Q fever is an emerging zoonosis caused by infection with Rickettsiae agent, *Coxiella burnetii* [1]. Q fever is characterized by fever with interstitial pneumonitis. Sixty percent of infected individuals are asymptomatic, but rare cases can develop fulminant hepatic failure and even acute renal failure [2],[3]. Cytomegalovirus (CMV) infection is typically a latent asymptomatic disease in otherwise healthy people. However, CMV-related hepatitis is common in immunocompromised patients, such as transplant recipients and neonates [4], and can also occur in immunocompetent patients [5],[6]. Herein, we report an immunocompetent man infected with *C. burnetii* and CMV who presented with fulminant hepatic failure and acute renal failure.

## Case presentation

A 53-year-old male farmer who bred geese, fowls, dogs, and cats, and had frequent contact with their carcasses presented at our institution with intermittent fever for the previous week in July of 2013. He reported taking anti-histamines for his urticaria in recent years. His symptoms included general malaise, icteric sclera, yellowish skin, bilateral plantar skin rash, and a headache in the temporal and frontal areas. He denied any history of travel, unprotected sex, insect bites, transfusion, or toxin exposure.

**Figure 1:**
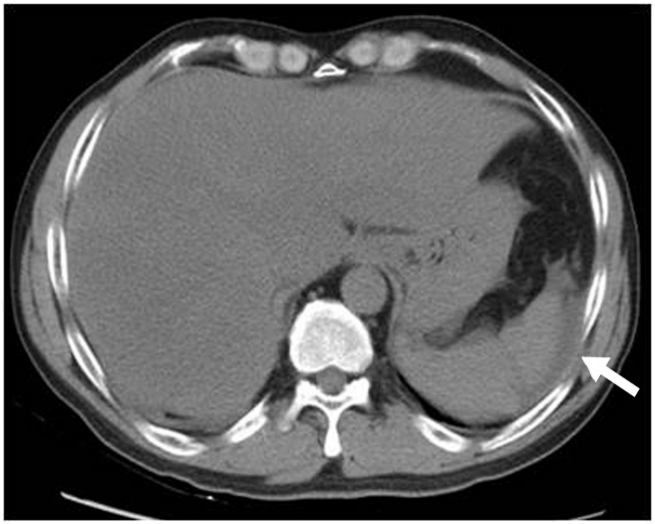
**Non-contrast computed tomography of the abdomen on admission showed generally decreased liver parenchymal attenuation and mild ascites (arrow), compatible with acute hepatitis.**

On admission, his body temperature was 39.1°C, pulse rate was 110 beats per minute, and blood pressure was 121/82 mmHg. He was oriented but had mild agitation. He had icteric sclera but no Kayser-Fleischer ring; his abdomen was not tender, and he had yellowish skin with several linear and erythematous lesions over bilateral plantar areas, but no eschar-like lesions. The results of a neurologic examination were unremarkable. A hemogram indicated a leukocyte count of 11,770/μL with 13% monocytes, and a platelet count of 85,000/μL. The prothrombin time was 19.1 s (reference range: 8-12 s) and the activated partial thromboplastin time was 36.2 s (reference range: 23.9-35.5 s). Biochemical analysis indicated elevated aspartate aminotransferase (2561 IU/L), alanine aminotransferase (2263 IU/L), alkaline phosphatase (274 IU/L), gamma glutamyltransferase (824 IU/L), total bilirubin (7.2 mg/dL), direct bilirubin (6.2 mg/dL), urea nitrogen (30 mg/dL), creatinine (2.6 mg/dL), and C-reactive protein (4.23 mg/dL). Analysis of arterial blood gas indicated a pH of 7.439 (reference range: 7.38-7.42) and HCO_3_^-^ of 19.5 mEq/L (reference range: 22-28 mEq/L). A chest X-ray was unremarkable but an abdominal computed tomography indicated generally decreased liver parenchymal attenuation and mild ascites, compatible with a diagnosis of hepatitis (Figure 1).

He was treated empirically with parenteral penicillin G (3 MU every 8 h) and parenteral levofloxacin (500 mg every 24 h) under impression of Leptospirosis and gram-negative bacterial infection. However, his fever persisted and he became drowsy with asterixis on day 6. At this time, there were also increases in the total bilirubin (18.2 mg/dL), direct bilirubin (14.2 mg/dL), and serum creatinine (5.9 mg/dL). Levofloxacin was discontinued and parenteral ceftazidine (2 g every 24 h) and vancomycin (1 g every 72 h) were administered. However, his condition continued to deteriorate. On day 8, there were generalized dark-red, painless, non-blanching macules over his trunk and four limbs and his serum total bilirubin had increased to 26.5 mg/dL. Penicillin G was replaced by oral doxycycline (100 mg every 12 h). There was no bacteria growth in blood cultures, and the serum anti-HAV IgM, HBsAg, anti-HCV Ab, anti-EB-VCA IgM, anti-HIV antibody, anti-HSV IgM and IgG were negative. Ceruloplasmin, anti-smooth muscle antibody, and anti-nuclear Ab were within the normal ranges. The serum collected for Q fever and *Leptospira* on day 2 was negative. Polymerase chain reaction (PCR) for *C. burnetii* also showed negative. On day 10, the serum anti-CMV IgM test was positive and he was given parenteral gancyclovir, after which his consciousness, liver, and renal function improved.

On day 12, the phase II IgG for Q fever by immunofluorescent assay (IFA) was 1:320, and phase II IgM for Q fever was negative (performed at Taiwan Centers for Disease Control). According to the diagnostic criteria [7], the patient’s course was compatible with acute Q fever. Microscopic agglutination tests using serum antibodies against *Leptospira santarosai serovar shermani*, *L. borgpetersenii serovar poi*, and *L. tarassovi* (performed at Taiwan Centers for Disease Control) were negative. Parenteral levofloxacin (500 mg every 24 h) was reinitiated on day 21.

**Figure 2:**
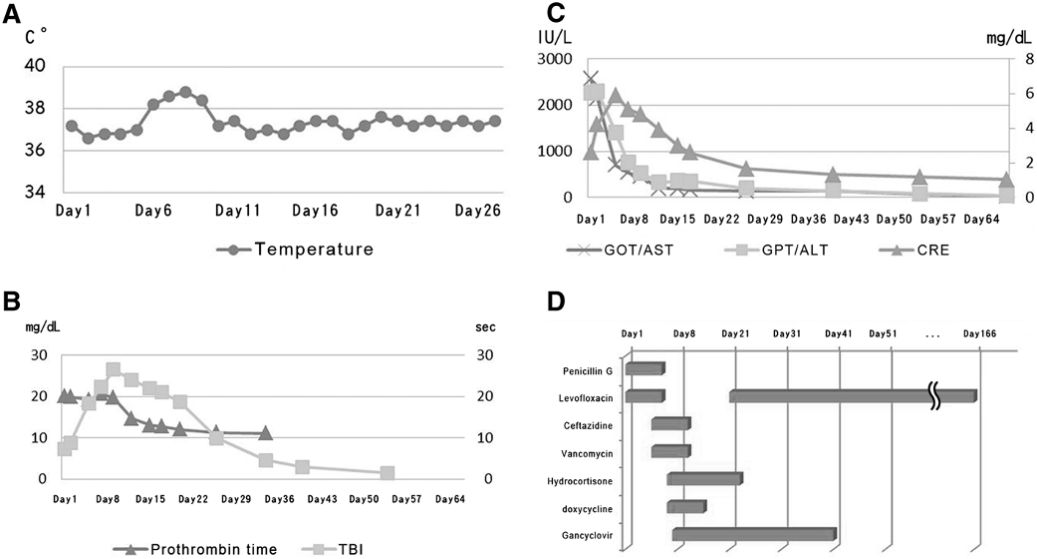
**Body temperature, laboratory and drug treatments during the whole course. A**: body temperature, **B**: prothrombin time and total bilirubin level (TBI), **C**: aspartate aminotransferase (AST), alanine aminotransferase (ALT), and creatinine, **D**: drug treatments.

On day 27, there were decreases in the serum total bilirubin (9.9 mg/dL) and creatinine (1.65 mg/dL) and the patient was discharged. After completion of a 24-week course of oral levofloxacin (500 mg every 24 h), there was normalization of the serum total bilirubin (0.8 mg/dL) and serum creatinine (1.04 mg/dL). A follow-up abdominal CT at 4 months indicated normal liver parenchymal attenuation without ascites. During the subsequent follow-up, he had not recurrent episodes or other discomfort. Figure 2 summarizes the clinical, laboratory and drug treatments.

## Conclusion

About 60% of patients with Q fever are asymptomatic, but patients with acute Q fever develop febrile episodes that are accompanied by atypical pneumonia (47% of cases), hepatitis (5% of cases), occasionally clinical hepatomegaly, and rarely jaundice [7]. A case report identified a patient with Q fever and granulomatous hepatitis [8]. In Taiwan, hepatitis was reported in 55% of patients with Q fever, and fulminant hepatic failure was rare [9]. Glomerulonephritis was recognized in chronic Q fever due to complications of endocarditis [10]. However, acute renal failure due to Q fever is rare [11]. In the present case, we identified a patient with Q-fever and CMV infection who presented with fulminant hepatic failure and acute renal failure.

The criteria for confirmation of acute Q fever is a titer of phase II IgM > = 80 or a fourfold increase of phase II IgG titers [7]. In our current case, the diagnosis of acute Q fever is based on the titer change of phase II IgG (from negative to 1:320 by IFA). However, the PCR of *C. burnetii* on 2^nd^ admission day and phase II IgM titer on 12^th^ admission day are both negative. A report from Taiwan Centers for Disease Control (TCDC) in 2011 used PCR and IgM IFA to check the serum of patients who diagnosed as acute Q fever by serology [12]. They found 29% patients with phase II IgM titer > = 80; 60% patients with positive PCR assay, and 78% patients with one of PCR and IgM titer > = 80. In other word, 71% patients are phase II IgM titer < 80; 40% patients have negative PCR; 22% patients have both negative. In our current case, it belongs to both negative. However, the use of PCR assay can actually facilitate the early diagnosis of acute Q fever.

Taiwan has many carriers of chronic hepatitis B and C [13], and the clinical manifestation of hepatitis due to acute Q fever is similar to those of patients with viral hepatitis. In addition, there is apparently no difference in replication of the hepatitis virus after acute Q fever hepatitis [14]. Thus, the presence of chronic hepatitis B and C infection seems unrelated to the development of Q fever symptoms.

CMV often co-infects patients with human immunodeficiency virus [15], pneumocystis jeroveci [16], herpes simplex virus [17], clostridium difficile [18], Epstein- Barr virus [19], and those with acquired immunodeficiency syndrome or who use immunosuppressants. In the present case, fulminant hepatic failure and acute renal failure occurred simultaneously. These symptoms are suggestive of Weil’s disease, caused by *Leptospira* [20],[21], which we were able to exclude. We later confirmed a diagnosis of Q fever. The causative microbe (*C. burnetti*) should be sensitive to levofloxacin, but the patient did not respond to the initial therapy of penicillin and levofloxacin, and his jaundice only subsided after use of gancyclovir. This indicates an important role of CMV infection in this case. However, CMV infection is latent in healthy people, and severe hepatitis only occurs in immune-compromised patients [4]. Previous reports indicated that CMV infection occurs in 0 to 36% of critically ill patients, mostly at 4 to 12 days after ICU admission [22]. Most cases with CMV infection present with pneumonia, and a small number present with liver involvement [5],[6]. In addition, cases with CMV-related hepatitis often present with mild elevations of liver enzymes and bilirubin, and only rarely with fulminant hepatic failure [4]. In the present case, we could not determine if acute hepatic failure due to Q fever reactivated the latent CMV, or if a reactivated CMV infection precipitated acute hepatic failure and Q fever.

In conclusion, co-infection with CMV and *C. burnetti* can cause acute hepatic and renal failure, with symptoms that mimic Weil’s disease (Leptospirosis). CMV co-infection should be considered in patients who present with Q fever when there is no response to the standard antimicrobial agents used against *C. burnetti*.

## Consent

Written informed consent was obtained from the patient for publication of this case. A copy of the written consent is available for review by the editor of this journal.

## Authors’ original submitted files for images

Below are the links to the authors’ original submitted files for images. Authors’ original file for figure 1 Authors’ original file for figure 2
