# Supplementary figures and images for: Fulminant hepatic failure and acute renal failure as manifestations of concurrent Q fever and cytomegalovirus infection: a case report
Source: BMC Infect Dis. 2014 Dec 9;14:651. doi: 10.1186/s12879-014-0651-8 (PMC4264321; doi:10.1186/s12879-014-0651-8)

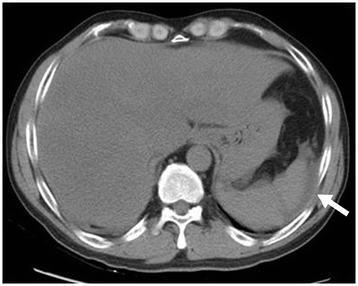

Supplement: Supplementary file 1 — Authors’ original file for figure 1 [file 12879_2014_651_MOESM1_ESM.gif]

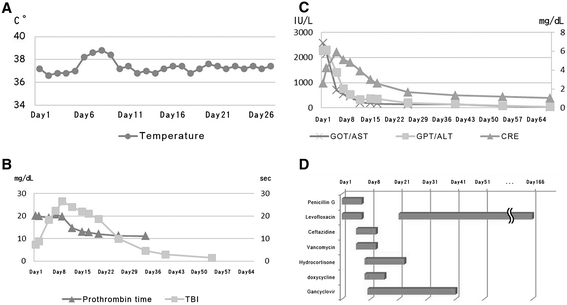

Supplement: Supplementary file 2 — Authors’ original file for figure 2 [file 12879_2014_651_MOESM2_ESM.gif]
